# Chronic stress, behavioral tendencies, and determinants of health behaviors in nurses: a mixed-methods approach

**DOI:** 10.1186/s12889-022-12993-5

**Published:** 2022-03-30

**Authors:** Luis Heuel, Svea Lübstorf, Ann-Kathrin Otto, Bettina Wollesen

**Affiliations:** 1 Department of Biopsychology and Neuroergonomics, Technical University of Berlin, Fasanenstr. 1, 10623 Berlin, Germany; 2 Faculty of Psychology and Human Movement Science, University of Hamburg, Turmweg 2, 20148 Hamburg, Germany

**Keywords:** Nurses, Health behavior, Occupational stress, Social determinants of health

## Abstract

**Background:**

Nurses experience high, and often chronic, levels of occupational stress. As high-quality care requires a healthy workforce, individualized stress-alleviating interventions for nurses are needed. This study explored barriers and resources associated with health behaviors in nurses with different stress levels and work-related behavioral tendencies and identified health behavior determinants based on the Health Action Process Approach (HAPA) model.

**Methods:**

Applying a mixed methods transformative triangulation design, *n* = 43 nurses filled out chronic stress (SSCS) and work-related behavior and experience patterns (German acronym AVEM) questionnaires, and participated in semi-structured interviews. With content analysis, categories of health behavior-related barriers and resources emerged. Behavior determinants (self-efficacy, outcome expectancies), health behavior, and barriers and resources were quantified via frequency and magnitude coding and interrelated with SSCS and AVEM scores to link level of health behavior with potential influencing factors. Nonparametric tests explored differences in quantified variables for SSCS and AVEM scores and 4-step-hierarchical regression analysis identified predictors for health behavior.

**Results:**

Eighty-four percent of the nurses were chronically stressed while 49% exhibited unhealthy behavioral tendencies at the workplace. 16 personal and organizational themes (six resources, ten barriers) influenced health behaviors. Stress was associated with resource frequency (*p* = .027) and current health behaviors (*p* = .07). Self-efficacy significantly explained variance in health behaviors (*p* = .003).

**Conclusion:**

Health promotion related barriers and resources should be considered in designing nurse health promotion campaigns. Practitioners need to individualize and tailor interventions toward stress and behavioral experiences for sustainable effects on adherence and health.

**Supplementary Information:**

The online version contains supplementary material available at 10.1186/s12889-022-12993-5.

## Background

Demographic changes and medical advances necessitate functioning health care systems [1]. However, nursing shortages across European countries are apparent, with the potential for substantial health care crises [2]. The shortage of skilled personnel is associated with increased work demands and (chronic) stress for nurses [3]. Excessive working hours, low perceived job control, and lack of workplace social support have been found to be predecessors of nurse stress [4–6]. Stress experiences, in turn, may lead to absenteeism, burnout symptoms and job turnover [7, 8]. Breaking this vicious cycle of staff shortages, increasing work demands, and job turnover is one of the major challenges of the upcoming decades.

One way to approach the problem of nursing shortage is to improve the health of the current nursing workforce by reducing perceived stress [9]. According to Lazarus’ transactional theory [10], stressors in the environment are interpreted by an individual, and, depending on personal and contextual resources, a secondary appraisal might induce stress. Thus, the same objective stressor may activate a different stress response in different individuals [11]. If resources are abundant, reduced detrimental stress effects may ensue [12]. Environmental resources include organizational characteristics such as job security and control, participation opportunities, feedback and coworker and supervisor support [13]. Personal resources reflect personality traits, attitudes, knowledge, and behavioral tendencies of an individual. For instance, problem-focused coping [14], and trait mindfulness [15] have been identified as health-enhancing resources.

In line with transactional theory, nurses differ in stress perception and general and mental health [16]. Environmental and personal resources may protect against health-impairing consequences of stress and burnout [17] and improve work engagement [18]. Further, health behaviors (e.g., physical activity, healthy dieting) have been found to reduce the perception and negative consequences of stress [19]. Therefore, strengthening personal and organizational resources at the workplace may alleviate health consequences of stress for nurses.

Differences in stress perceptions may be linked to employing different stress coping strategies to deal with workplace adversity [20]. These coping strategies including, e.g., offensive problem coping, social support seeking, or avoidance, are linked to workplace behavior tendencies [21]. Schaarschmidt and Fischer [22] developed the Arbeitsbezogene Verhaltens- und Erlebensmuster (AVEM) questionnaire assessing *work-related behavior and experience patterns*. The AVEM evaluates job resources and coping tendencies of respondents and estimates risky behavior styles. AVEM has been applied with different settings and populations. For instance, it has been shown that a high proportion of German High school teachers exhibited high-risk work behaviors [23]. Studies investigating the relationships of AVEM with health-related mechanisms in nursing populations found associations with sense of coherence [24], physical and psychological health [25], and perceived work burdens and stress management strategies [3]. Wollesen et al. [3] concluded that health promotion interventions should be conceptualized in a way that individual resource perception and coping styles are accounted for.

Interventions aiming for resource enhancement in nurses have been investigated extensively [26–28]. For instance, physical activity (PA) decreases perceived stress [29], reinforces personal and social resources to cope with occupational stress [30], and promote the ability to deal with situational stressors [31]. Moreover, resource gains can be accomplished with cognitive-behavioral interventions such as mindfulness-based stress reduction (MBSR) programs. MBSR was found to improve stress perceptions, anxiety symptoms, work satisfaction, and resilience in nursing populations [32–35].

Despite evidence for the efficacy of interventions, nurse health is generally poor. One reason for this may be the time-bound nature of health promotion interventions. Typically, interventions across health behaviors are carried out for a finite time period which limits the potential for sustained behavioral change [36]. Furthermore, personal and/or organizational barriers may impede continuous health behaviors when long-term health promotion programs are available [37]. Barriers for nurses to participate in health promotion measures include organizational aspects such as limited time resources, interpersonal factors, e.g., pressure from coworkers and supervisors, and intrapersonal characteristics such as the aspiration to deliver high-quality care [38].

Another consideration for poor nurse health is the paucity of the assessment of health behavior-related cognitive determinants in health promotion interventions [36]. Cognitive determinants are psychological mechanisms that mediate the relationship between antecedent factors (e.g., sociodemographics, beliefs) and behavior [39]. Various social cognition models have been brought forward that attempt to explain behavior with cognitive determinants. For instance, as one of the most prominent behavior determinants, Bandura [40] first brought forward the concept of self-efficacy. Self-efficacious individuals believe in their abilities to accomplish goals [41] and more reliably adopt and maintain health behaviors [42]. Self-efficacy is included in other social cognition models such as the Protection Motivation Theory [43]. In the Theory of Planned Behavior [44], perceived behavioral control is operationalized similarly to Bandura’s concept of self-efficacy. The Health Action Process Approach (HAPA) [45] identifies self-efficacy as an important motivational and volitional predictor of behavior.

Another recurring determinant in different models of behavioral change are outcome expectancies, or costs and benefits of engaging in a behavior [46]. Similarly to self-efficacy, there are various terms for outcome expectancies in different models (response costs; behavioral beliefs; ‘pros and cons’ of behavior adaption [39, 47]).

Lastly, the HAPA and social cognitive theory take into account barriers and resources as influencing factors for behavior [48, 49]. However, little research has examined nurse-specific barriers and resources which may influence individual health behaviors.

### Aims and objectives

The aim of the current study is to explore nursing-specific barriers and resources for health behavior and to identify predictors of nurse health behaviors. In the long-term, the present study may inform a need-tailored app to alleviate stress and promote health in nurses (acknowledged in Fundings).

The objectives of the study are fourfold: (1) qualitative assessment of health behavior determinants, (2) quantitative assessment of stress levels and behavioral tendencies, (3) exploration of barriers and resources for health behaviors in nurses, and (4) identification of predictors for health behaviors. This approach may elicit behavioral and contextual factors linked to health behavior-specific self-efficacy and outcome expectancies in nurses [50]. Additionally, participants are not constrained by a priori questionnaire categories which may limit the response width of participants [51].

Five research questions were guiding this study:

1. *What are the stress levels and work-related behavior patterns of nurses?*
2. *What are barriers inhibiting and resources facilitating health behaviors in nurses?*
3. *What are the magnitudes of health behavior-specific self-efficacy and outcome expectancies and current level of health behavior?*
4. *What are associations of stress levels and AVEM patterns with determinants of health behavior (self-efficacy, outcome expectancies, barriers and resources), and current health behaviors?*
5. *In how far do stress scores, AVEM patterns, barrier and resource frequency, self-efficacy, and outcome expectancies explain health behaviors?*

## Materials and methods

### Study design

This study had a concurrent cross-sectional, mixed-methods design. We conducted cross-sectional survey and semi-structured interviews. Reporting adhered to the guidelines of Levitt et al. [52]. Qualitatively, the study systematically established categories and themes of nurse-specific barriers and resources for health behaviors. With data transformation, interview statements were quantified and triangulated with the survey data [53] to examine stress, behavioral tendencies and self-portrayed health determinants as potential predictors of health behavior. Data collection took place from February through May 2020. Ethical approval was received by Technical University of Berlin’s Ethics Committee (WO_02_20200117).

### Participants and setting

A convenience sample of *N* = 93 nurses from different settings (hospital, geriatric, and outpatient care) participated in the quantitative part of the study. The various settings were chosen to represent different job contents and obstacles of the daily work hustles. Of the 93 participants who took the survey, *n* = 43 (*n* = 10 outpatient care, *n* = 16 geriatric care, and *n* = 17 from different hospital departments such as emergency, intensive care, cardiology, rehabilitation) consented to participate in a follow-up interview. This equals a response rate of 46% for all study parts. The mean age of nurses was 40.21 years (SD = 13.27). Inclusion criteria were employment as a nurse, at least 18 years of age and participation in both the quantitative and qualitative segments of the study. Non-nursing employees were (e.g., cleaning and kitchen personnel, janitors, non-nursing managers) excluded from the study.

### Materials

#### Chronic stress

For stress assessment, the 12-item Screening Scale for Chronic Stress (SSCS) of the Trier Inventory for Chronic Stress (TICS) was applied [54]. Cronbach’s α ranges from 0.84 to.91 which indicates good to very good internal consistency [55]. Respondents are categorized as either not chronically stressed (score < 15) or chronically stressed (score ≥ 15). Items include stress-related statements such as ‘There are times in which I have to fulfil too many duties’. Respondents indicate in how far the statements apply to them on a 5-point Likert scale (never – very often). Due to the high incidence of chronic stress in nurses, the SSCS is an appropriate measurement tool for the purpose of the study.

#### Work-related behavior and experience

The AVEM-44 (German: Arbeitsbezogene Verhaltens- und Erlebensmuster) [56] constitutes a shortened version of the original 66-item version of the AVEM questionnaire, identifies three areas of work-related behavior and coping styles, namely work commitment, resilience, and emotional well-being, partitioned into 11 dimensions (subjective importance of work, professional ambition, readiness to overexert, strive for perfection, distancing ability, resignation tendency, offensive problem coping, mental balance, professional success, life satisfaction, and social support experiences). Respondents are classified into one of four patterns of behavioral tendencies including:

1. Pattern G – Health: The most desirable pattern expresses itself via high, but non-excessive work engagement. Usually, subjective importance of work, readiness to overexert, and strive for perfection are slightly elevated, despite a high distancing ability. Resilience values are typically increased and the same applies to occupational emotional stability.
2. Pattern S – Conservation (of resources): Individuals with this pattern tend to conserve their available resources and thus exhibit low work engagement. However, relatively high values of distancing ability and mental balance are maintained, as well as high life satisfaction which may be achieved by recreational and/or social activities outside the occupation.
3. Risk pattern A – Overexertion: Workers with this pattern may exhibit unhealthy high work engagement. Thus, subjective importance of work, strive for perfection, and readiness to overexert are drastically increased. The most pronounced difference to other patterns is the inability to distance oneself from work-related issues. Further, negative emotions are recurring. Overall, high effort is not accompanied by a corresponding level of work-related reward. Oftentimes, individuals are unable to relax and are at increased risk of coronary heart disease.
4. Risk pattern B – Resignation: The most prominent indicator for this pattern is a heightened resignation tendency, paired with low values on offensive problem coping, mental calmness and balance as well as job and life satisfaction. On the dimensions work engagement, subjective importance of work, and career ambitions, pattern B individuals score, similar to pattern S individuals, low. However, in contrast to pattern S, resigning individuals are less able to distance themselves from work. Importantly, behavioral and experiential tendencies are similar to burnout symptoms.

The scale consists of four items per dimension presented with a 5-point Likert scale (applies not at all – applies perfectly). Via cluster analysis, respondents are allotted one of the four patterns.

Validity is supported by good agreement between AVEM and related constructs (Maslach Burnout Inventory, Big-Five List). Furthermore, good reliability has been demonstrated for the scale, with internal consistency ranging from 0.75 to 0.83. [22].

#### Interviews

Interviews contained semi-structured questions pertaining to.

1. work stress;
2. utilization of occupational health promotion programs and
3. health behavior determinants including self-efficacy and outcome expectancies, and current health behavior.

Regarding (3), the interview contained questions related to health behavior-specific self-efficacy (‘How do you estimate your personal confidence to perform health behaviors in the future?’), outcome expectancies (‘What would change for you personally if you participated in health promotion programs?’ [If answer was one-sided: ‘Would something improve/worsen?’]), and about current health behaviors (‘Have you lately done something for your health?’ [If yes: ‘What health behaviors have you engaged in? How often per week? How long per session?’] [If no: ‘Do you think about engaging in health behaviors in your future?’]). Interviewers informed participants about confidentiality and encouraged them to respond truthfully. The interview did not include questions about risk perception due to its minor contribution in explaining intention variance [57]. Interviews were transcribed by independent student assistants.

For the goal of identifying health behavior-specific barriers and resources, interview topics (1) – (3) were searched.

The Supplementary Materials contain example statements for self-efficacy, outcome expectancies, and health behavior, as well as a description with identified themes for barriers and resources.

### Procedure

#### Quantitative data collection

The research team (L.H., S.L., and A.-K.O) visited and surveyed participants from an outpatient nursing facility and a nursing home at their work sites in Germany in February 2020. After signing informed consent, nurses filled in questionnaires including basic demographics as well as the SSCS and AVEM-44 at work. As data collection took place during the emerging Covid-19 crisis, remaining participants completed an online version of the questionnaires administered via the software *LimeSurvey*. Completion of the survey took approximately 15 min and participation was reimbursed with 10€.

#### Interviews

At the end of the questionnaire, respondents received an invitation to participate in a follow-up interview. Informed consent and study information included the qualitative part, with the remark that there is no obligation to participate in the interview. A few days after participants took the survey, nurses who consented to participate were contacted by the research team via telephone and, respecting participants’ work schedule, appointments for the interview were arranged during leisure time. Authors L.H., S.L., and A.-K.O. conducted the interviews. It was ensured that no personal relationship between researchers and participants existed prior to the interview, with the exception of the encounter during quantitative data collection. At the arranged time, interviewers contacted participants and repeated the study goals and clarified open questions. Duration of the interview was between 13 and 40 min. Interviews were audio recorded and subsequently transcribed verbatim. Respondents received 25€ for participation.

### Analysis

#### Quantitative analysis

Frequency distributions of stress levels and AVEM patterns were analysed. One-way ANOVA frequency analyses were conducted for the pooled relatively healthy patterns (G/S) vs the relatively unhealthy patterns (A/B) with respect to differences in stress experiences.

#### Qualitative analysis

L.H. and S.L. coded interview transcripts. First, health behavior barriers and resources were explored by searching the transcripts. We applied the methodology of deductive-inductive qualitative content analysis [58], by first defining barriers and resources. Building on the work of Gutsch et al. [59], we defined resources a priori as any personal and/or organizational factors that may increase resilience toward work demands and reduce negative heath consequences of job stress. Barriers were operationalized as producing an opposite effect. Categories within these definitions were established inductively. L.H. developed an initial coding frame with preliminary barrier and resource categories by identifying categories in a subset of the transcripts. Subsequently, S.L. independently applied the coding frame to the same subset. Inconsistencies were resolved by discussion. When the coders agreed on the coding frame, L.H. independently coded the remaining transcripts. For the analysis, the software package *MaxQDA AnalyticsPro 2020* was used.

In the first coding cycle, L.H. coded self-efficacy, outcome expectancies, and current health behaviors. We coded only the segments that directly followed the related interview question (see Materials).

#### Data transformation

The qualitative assessment from the first cycle was transferred in a numerical magnitude scheme in a second coding cycle. L.H. and S.L. assessed the magnitude of self-efficacy, outcome expectancies, and current health behavior [60]. Accordingly, each testimony was assigned a magnitude score for self-efficacy (1 = very low – 5 = very high), outcome expectancy (1 = negative – 5 = positive), and current health behaviors (1 = very poor – 5 = very good). The coding team discussed any discrepancies until consensus was reached.

Also, L.H. assigned frequency scores for barriers and resources, respectively. For instance, a transcript that yielded a total of two health behavior-specific barriers and one resource would result in barrier frequency = 2, and a resource frequency = 1. For the transformative interview analysis, we again used *MaxQDA AnalyticsPro 2020*.

#### Mixed-methods analysis

Stress and AVEM outcomes were triangulated with the magnitude scores of self-efficacy, outcome expectancy, and current health behaviors, as well as with barrier and resource frequency scores. Next to chi^2^ frequency analyses, we compared chronically stressed vs not chronically stressed participants regarding differences in magnitude of health behavior determinants and barrier and resource frequency scores with non-parametric Mann–Whitney U tests. The same comparisons were applied for the AVEM patterns using the non-parametric Kruskal–Wallis test. Next, Spearman correlational analysis was conducted among the AVEM patterns, stress scores, magnitude scores of health behavior determinants, and barrier and resource frequency scores. Finally, we performed a 4-step hierarchical regression analysis with health behavior as the dependent variable to explore which variables explain individual health behavior. Below is a summary of the 4 steps of the regression analysis:

- Block I: AVEM patterns
- Block II: SSCS scores
- Block III: Barrier Frequency, Resource Frequency
- Block IV: Self-efficacy, Outcome Expectations

We used IBM SPSS 25.0 for all quantitative analyses.

## Results

### Characteristics of stressed vs non-stressed nurses

Results are reported for the nurses that responded to all study parts. Table 1 summarizes our findings in relation to stressed vs not stressed participants. Of the *n* = 43 nurses, *n* = 36 were identified as being chronically stressed. Further, *n* = 14 participants were affiliated with the healthy pattern G of the AVEM, eight nurses with pattern S. Risk pattern A was the most prevalent, with *n* = 15 participants exhibiting unhealthy dedication to their employment. Lastly, *n* = 6 participants showed characteristics of burnout symptoms (risk pattern B). One-way ANOVA showed that stress levels were significantly lower for relatively healthy (G/S) vs. relatively unhealthy (A/B) patterns (*F(1,41)* = *6.950, p* = *0.012, Eta*^*2*^ = *0.145*). Occupation, health behavior determinant magnitudes, and barrier and resource frequencies are reported in Table 1 for stressed vs not stressed groups (see Supplementary Material for exemplary statements of different magnitudes assignments).

**Table 1:** Sociodemographics and characteristics of stressed vs non-stressed nurses

Variables	Stress Level		Total N (%)
	**chronically stressed**	**not chronically stressed**	
Count	36	7	43 (100)
Male	8	2	10 (23)
Female	28	5	33 (77)
Age (SD)	40.7 (13.6)	37.4 (12.2)	40.1 (13.27)
**Leading position**			
yes	9	2	11 (26)
no	27	5	32 (74)
**Nursing Occupation**			
Hospital	12	5	17
Geriatric	14	2	16
Outpatient	10	0	10
**AVEM Patterns**			
G Pattern	12	2	14 (33)
S Pattern	4	4	8 (18)
A Pattern	14	1	15 (35)
B Pattern	6	0	6 (14)
**Barriers & Resources**^a^	Stressed mean (SD)	Not stressed mean (SD)	Total mean (SD)
Resources	2.28 (1.26)	3.86 (1.77)	2.53 (1.45)
Barriers	2.47 (1.42)	1.57 (0.78)	2.33 (1.37)
**Health promotion determinant**^b^			
Self-efficacy	3.89 (1.09)	3.86 (1.95)	3.88 (1.23)
Outcome expectancies	4.03 (1.05)	3.43 (1.13)	3.93 (1.08)
Health promotion activities	2.94 (1.22)	3.86 (1.07)	3.09 (1.23)

^a^Frequency score

^b^Magnitude score

### Barriers and resources: identified themes

*C*ontent analysis revealed 16 themes for nurse-specific health behavior-related barriers and resources within the superordinate categories:

Personal. Four resource categories (dispositional character traits, team social support, private social support, private compensation) and six barrier categories (dispositional character traits, sleeping problems, team social support, dieting and smoking, domestic duties, and injury/illness) were identified. In total, there were *n* = 93 counts for resources, *n* = 48 counts for barriers.

Organizational. The analysis identified two job resources (shift structure, occupational health promotion programs) and four work-related barriers (job demands, occupational health promotion logistics, occupational health promotion attractivity, work site-residence distance). In total, the nurses mentioned organizational resources *n* = 16 times, whereas barriers were reported *n* = 51 times (see Supplementary Material for example statements of barriers and resources).

### Associations between stress levels, barrier and resource frequency, and health behavior determinant magnitude scores

Resource frequency exhibited partial significance such that not chronically stressed participants had higher resource frequency (vs chronically stressed), χ^2^ = 12.218, *p* = 0.057, C = 0.47. Moreover, there was a significant difference in self-efficacy. Chronically stressed (vs not chronically stressed) participants had lower self-efficacy ratings (χ^2^ = 15.968, *p* = 0.003, C = 0.52). Lastly, chronically stressed individuals had significantly more positive outcome expectancy ratings compared with the non-stressed group (χ^2^ = 11.847, *p* = 0.019, C = 0.47).

Results of the Mann–Whitney U test indicated that resource frequency was lower for chronically stressed (Mdn = 2) than not chronically stressed (Mdn = 4) participants (U = 60.5, z = -2.22, *p* = 0.027). Further, current health behavior was higher for not chronically stressed (Mdn = 4) than for chronically stressed (Mdn = 3) individuals, however this result failed to be significant (U = 72.5, z = -1.81, *p* = 0.07).

### Associations between AVEM patterns, barrier and resource frequency and health behavior determinant magnitude scores

Analyses revealed no significant differences in frequency or magnitude scores for AVEM patterns, nor for relatively healthy (G/S) vs unhealthy (A/B) pooled patterns.

### Correlational analysis

The correlation matrix exhibited significant correlations between AVEM patterns, stress scores, determinants scores, and barrier and resource frequencies. Specifically, pattern G scores was negatively associated with overall barriers (rs = -0.332). Pattern S scores were negatively correlated with the SSCS score (rs = -0.469). Lastly, risk pattern A scores had a positive correlation with the SSCS score (rs = 0.312), and a negative correlation with health behaviors (rs = -0.315). Further, magnitude scores exhibited significant correlations among each other. Self-efficacy scores positively correlated with resource frequency (rs = 0.517) and with health behavior (rs = 0.691). Also, resource frequency had a significant positive correlation with health behavior (rs = 0.452), and a significant negative correlation with barrier frequency (rs = -0.337; Table 2).

**Table 2:** Spearman Correlation matrix

Variables	1	2	3	4	5	6	7	8	9
1. SSCS raw score	1								
2. G Pattern score	-.114	1							
3. S Pattern score	-.469**	-	1						
4. A Pattern score	.312*	-	-	1					
5. Resource Frequency	-.13	.243	.21	-.233	1				
6. Barrier Frequency	.199	-.332*	-.073	.107	-.337*	1			
7. Self-efficacy	-.187	.057	.142	-.219	.517**	-.115	1		
8. Outcome expectancies	.182	.218	-.089	.049	.238	.153	.237	1	
9. Health behavior	-.238	.124	.185	-.315*	.452**	-.204	.691**	.218	1

^*^: *p* < .05; **: *p* < .01

### Regression analysis

**Fig. 1:**
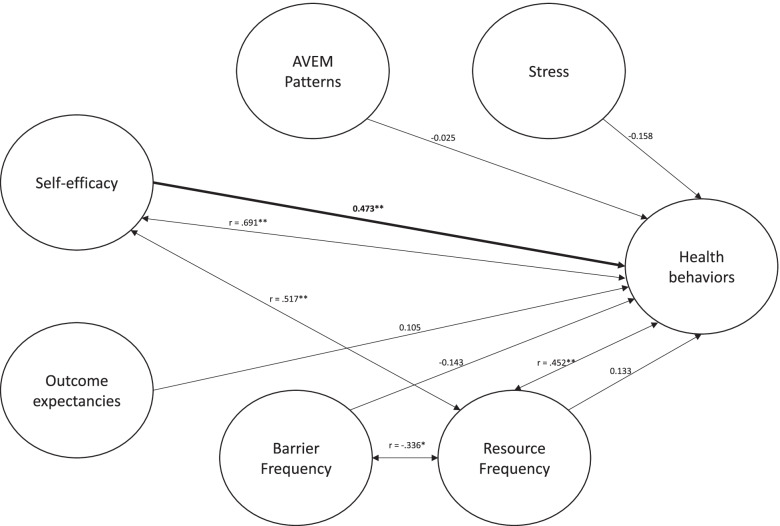
Mixed methods model of nurse health behavior, inspired by HAPA. Double arrows represent significant correlations between variables. Single arrows are standardized β of the last step regression analysis. *: *p* < .05; **: *p* < .01

The regression model (Table 3), exhibited significance in steps three (F(4, 38) = 3.135, *R*^2^ = 0.248, *p* = 0.025) and four (F(6, 36) = 4.886, *R*^2^ = 0.449, *p* = 0.001). In steps one and two, neither AVEM patterns nor stress scores were significant. Step three revealed a significant effect of resource frequency (t(38) = 2.596, *p* = 0.013). In step four, a significant effect of self-efficacy was found (t(36) = 3.225, *p* = 0.003) while resource frequency failed to be significant. Figure 1 shows a working mixed-methods model of nurse-specific health behaviors.

**Table 3:** Hierarchical regression analysis

Variables	Step 1		Step 2		Step 3		Step 4	
	**B**	**β**	**B**	**β**	**B**	**β**	**B**	**β**
Block I								
AVEM Pattern	-0.086	-0,075						
Block II								
AVEM Pattern			-0.003	-0.002				
SSCS raw score			-0.039	-0.258				
Block III								
AVEM Pattern					0.055	0.049		
SSCS raw score					-0.025	-0.169		
Resource frequency					0.328*	0.387		
Barrier frequency					-0.111	-0.124		
Block IV								
AVEM Pattern							-0.029	-0.025
SSCS raw score							-0.024	-0.158
Resource frequency							0.113	0.133
Barrier frequency							-0.128	-0.143
Self-efficacy							0.47**	0.473
Outcome expectancies							0.12	0.105
R^2^	.006		.0067		.248*		.449**	

B: Beta (non-standardized coefficient); ß: Beta (standardized coefficient); R^2^: coefficient of determination; *: *p* < .05; **: *p* < .01

## Discussion

This cross-sectional mixed-methods study used a multidimensional triangulation design with quantitative and qualitative data with the aim to shed light on the relationship between chronic job stress, and behavioral inclinations with nurse-specific determinants of health behavior. In the quantitative data collection, nurses of different work settings filled in the SSCS and AVEM questionnaire. Subsequently, they answered semi-structured interview questions in relation to health behavior-related barriers and resources as well as theoretically derived health behavior determinants. With qualitative content analysis, barriers and resources were categorized. Further, participants received magnitude scores regarding determinants of health behavior. In the triangulation phase, frequencies of barriers and resources and magnitudes of determinants were associated with stress levels and work-related behavior and experience patterns. The approach allowed for the proposition of a mixed-methods model of nurse health behavior, based on the HAPA model of behavioral change [61].

In the study sample, the majority (84%) of nurses was chronically stressed. The stress levels depict the severity of the current health care crisis, as previous studies with nurses found less extreme stress levels [3]. Stress perceptions may be linked to behavioral differences. In this study, those nurses with non-chronic stress exhibited healthier work-related behavior and experiences. This finding highlights the necessity to design interventions with varying contents for people with different behavioral tendencies [62].

The current study explored health behavior barriers and resources for nurses with qualitative content analysis. Semi-structured interviews were searched for any potential barriers and resources, subdivided into personal and organizational categories. There was a remarkable difference in the number of barriers and resources mentioned for the subdivisions. Across the sample, there were 93 (16) counts of personal (organizational) resources vs. 48 (51) personal (organizational) barriers. This finding has strong implications for future health promotion endeavors. If personal resources are available, interventionists and stakeholders need to decrease organizational barriers that inhibit nurse health behavior (e.g., fitting occupational health promotion programs with nurses’ work schedules, and ensuring that programs match the interests of nurses). Interventions typically do not consider organizational barriers sufficiently, which may be one reason for overall low intervention success in nursing [36]. Thus, despite good intervention approaches, for instance eHealth interventions with individualized modules [63], sustainable health promotion activities may not be achieved because organizational barriers are neglected. This stance is supported by McLean et al. [64] who argue that ‘further research to increase basic understanding of the factors, which act as a barrier to […] adherence, could facilitate development of strategies to overcome non-adherence’.

Organizational barriers included *occupational health promotion logistics, occupational health promotion attractivity, high job demands,* and *worksite-residence distance*. Thus, nurses may be more prone to engage in health behaviors if their company facilitates a health-promoting lifestyle. Facilitators may be higher levels of participation opportunities [37] and better shift working structures [65]. In line with this, Chesak et al. [66] argue that interventions should be complemented by changes in the work environment of nurses. Regarding resources, the most prominent theme was *private compensation*. Types of compensations included exercising, gardening, reading, and healthy cooking. The findings may be applied to health promotion interventions for nurses by alleviating barriers during the change process and enhance perceived resources.

In the next step, we triangulated the qualitative and quantitative data. Relationships of stress scores and AVEM patterns with health behavior determinant (self-efficacy, outcome expectancies, health behavior) magnitudes and barrier and resource frequency were analysed. Chronically stressed and not chronically stressed nurses differed in several aspects. Concerning self-efficacy, despite a higher mean rank for non-stressed participants (24.71 vs. 21.47), it was not significantly different in the groups when considering the non-normal distribution of stress scores. Nonetheless, self-efficacy remains one of the most important moderators of healthy stress coping [67]. Also, self-efficacy predicts nurse health by mediating the relationship between social support and resilience [68]. There are inconsistencies in the literature about the mechanisms of action that self-efficacy exerts on health behavior. For instance, general self-efficacy might moderate effects of job stress and work ability [69]; or self-efficacy may mediate an intervention effect on perceived stress [70]. However, given the manifold studies indicating evidence for positive effects of self-efficacy on health outcomes, it should remain a focal point of health promotion interventions.

Chronic stress was linked to higher numbers of mentioned barriers than non-chronic stress (mean rank 23.24 vs 15.64), however, the difference failed to be significant. This finding indicates that perceived barriers differ from objective (i.e., organizational) barriers. Accordingly, individual differences may determine perceptions of health behavior barriers, and barrier perception may be a fruitful target for upcoming health promotion interventions. Resource frequency, on the other hand, differed significantly between groups. Non-chronic stress was significantly related to higher resource frequency compared to chronic stress (mean rank 31.36 vs 20.18). This finding is in line with Lazarus’ [10] transactional theory of stress, which constitutes that insufficient personal resources will evoke stress perceptions. Strengthening perceived resources may therefore be an essential part of future health promotion programs for nurses.

We found health behavior to differ between chronically stressed and non-chronically stressed participants, however not reaching significance (*p* = 0.07). Previous research has shown that experiencing stress is often associated with decreased physical activity [71]. Stressed individuals may also engage in more health-impairing activities such as binge eating, meal skipping or smoking [72]. This finding, despite non-significance, is plausible as research has shown the regulatory effects of health behaviors such as healthy dieting and exercising on stress coping [73].

Outcome expectancies, defined as the balance between the pros and cons for changing current health activities, differed between the stress groups insignificantly (mean rank 23.14 vs 16.14). Outcome expectancies are an important determinant in various models of behavioral change such as the HAPA model [48] or social-cognitive theory [74]. One may therefore conjecture that non-stressed individuals, who tend to be more physically active [71], have more favorable outcome expectancies. Contrary to that, Lippke et al. [75], researching stage effects with the HAPA model, found that non-intenders scored *lower* on *cons* than both intenders and actors. This could indicate that individuals in the early motivational stage are principally aware that disadvantages for adopting a healthier lifestyle are sparse. Actors, on the other hand, may visualize vividly how pursuing a higher level of a given behavior may be linked to more disadvantages (i.e., higher expenditure of financial/social/cognitive resources). However, this is speculative, and more research is needed to elucidate these preliminary findings.

Finally, we established a model of nurse health behavior within our mixed-methods framework (Fig. 1). Our 4-step regression model revealed that, after controlling for AVEM patterns, stress scores, outcome expectancies, and sum scores of barrier and resource frequencies, self-efficacy significantly predicted health behaviors. This adds to the plentiful research on the positive impact of self-efficacy on health outcomes. Before inclusion of self-efficacy, resource frequency significantly predicted health behavior. As self-efficacy and resource frequency significantly correlated with each other, there may be collinearity between the two. This may be in line with the notion that different facets of self-efficacy are important resources in their own regard. Due to its substantial effects [76], health promotion interventions should aim to improve self-efficacy perceptions. As phase-specific self-efficacy is necessary throughout the complete change process [48], more research is needed to develop phase-tailored self-efficacy interventions.

### Strengths and limitations

A major strength of the present study is the mixed-methods design. By triangulating questionnaire and interview data, a more holistic understanding of nurse health can be established. The approach allowed for an analysis of the impact of barriers and resources on health behavior. Another strength is the open interview approach. Participants were encouraged to mention any thoughts concerning their own health activities, self-efficacy, and outcome expectancies. They were not limited by questionnaires that typically guide answers in a predetermined direction. Furthermore, the number of participants was rather large for a qualitative approach which typically involve less participants.

One limitation refers to participant selection. For instance, the work of hospital nurses may differ substantially between departments and thus, occupational resources and barriers vary markedly within and between hospitals. Also, while our results enlighten health behavior-related factors for German nurses, they may not be generalizable to the workforce in other countries. Nevertheless, nurses across the globe are facing precarious working conditions and stress.

Further, as the methodology for exploring barriers and resources was inductive, the results should be interpreted carefully. It is possible that we missed important barriers or resources that play a role in nurse health promotion. In a similar fashion, the study does not allow to make inferences about *qualitative* differences of barriers and resources. It is highly likely that not only the number of barriers and resources are linked to health outcomes, but different barriers influence health behavior differently.

Concerning the quantitative part of the study, time pressure and job demands may have influenced survey answers. However, as we aimed to depict work-related stress, this problem may be of minor importance.

Finally, the non-normal distribution of AVEM patterns made it difficult to find associations with other variables. Typically, studies incorporating the AVEM questionnaire contain very large numbers of participants [3, 77].

## Conclusion

The current findings shed light on the specific barriers and resources linked to health behaviors in nurses. The study extended the knowledge of the impact that stress and work-related behavioral tendencies have on nurses with regard to promoting health. The results should thus be considered in the development of future interventions for nurse health promotion. Importantly, individual differences in stress perceptions, and work-related behavior should be considered in the conception of future health promotion interventions. While health promotion interventions for nurse health are highly relevant, the many organizational barriers to health promotion found in this study suggest the need for structural changes in the health sector, such as higher financial compensation and organizational adjustments in the working structure. Current policies do not provide the necessary incentives for nurses to avoid job turnover. Similarly, the social recognition of the nursing profession prevents younger generations to consider a career in the sector. However, the requirement for health care facilities to be profitable prevents organizations from making changes in that regard.

Health promotion practitioners may account for health promotion barriers specific to health care organizations to improve intervention efficacy. Future studies should shed more light on the relationship between specific barriers and resources and nurse health behaviors. Also, as the current study showed that various forms of resources and barriers exist, it would be a fruitful endeavor to explore the differential impact toward health behaviors, possibly by applying quantitative methodologies with larger sample sizes.

## Supplementary Information

**Additional file 1.**

## Data Availability

The datasets used and/or analysed during the current study are available from the corresponding author on reasonable request.
