# Supplementary material for: Chronic stress, behavioral tendencies, and determinants of health behaviors in nurses: a mixed-methods approach
Source: BMC Public Health. 2022 Mar 30;22:624. doi: 10.1186/s12889-022-12993-5 (PMC8967083; doi:10.1186/s12889-022-12993-5)
Supplement: Supplementary file 1 — Additional file 1. [file 12889_2022_12993_MOESM1_ESM.docx]

| A.1. Barriers & resources – themes, example statements (translated), and counts | | | | |
| --- | --- | --- | --- | --- |
| Resources | Category | Definition | Example statement | counts |
| Personal | Dispositional character traits | Personality factors facilitating healthy stress coping / learned skills facilitating healthy stress coping | On most days I am able to distance myself from work as soon as work is done. | 17 |
|  | Team social support | Social support experienced by supporting colleagues | Teamwork in our team is very good. We support each other constantly. We help each other constantly. | 26 |
|  | Private social support | Social support experienced by friends, family and the like | I meet up with people I like and that’s how I can actually relax. | 17 |
|  | Private compensation (e.g., sport, gardening, reading, healthy eating a.o.) | Private activities directly or indirectly supporting mental and/or physical health | What contributed most to my relaxation is my garden. I travel a lot. I can relax well while gardening and I regularly help out at the local fire brigade. | 33 |
| Organi-sational | Occupational health promotion programs | Usage of occupational health promotion courses / coaching / training | I indeed participated in an extra training on stress [within my company] | 13 |
|  | Shift structure | Factors that facilitate health behavior via health promoting working structures (e.g., adaptive shift models) | We have a very good shift structure in the hospital, so we do not have too much stress, which leaves enough room for sports, social activities, and so on. | 3 |
| Barriers | Category | Definition | Example statement | counts |
| Personal | Sleeping problems | Statements on sleeping problems, possibly but not necessarily caused by shift work | [I] have trouble falling asleep […] | 7 |
|  | Injury/illness | Pre-existing medical conditions inhibiting healthy stress coping | […] chronic leg pain for years, I have even been to the doctor, they couldn’t tell me what was wrong. | 6 |
|  | Dispositional character traits | Personality factors inhibiting healthy coping | I don’t believe I can change anymore, I guess for me, I won’t be able to do it any better. And I won’t get out of this situation. I’m probably too stuck in my habits already. | 4 |
|  | Dieting and smoking | Direct statements on unhealthy coping strategies; smoking/unhealthy eating | If it is getting too stressful, I go and grab a coffee outside. I smoke a cigarette. | 11 |
|  | Domestic duties | Domestic duties inhibiting behavioral change | We are currently in a learning phase, we recently have had a child. This can cause stress as well | 13 |
|  | Team social support | Non-supporting working team structures | I am this kind of person to take care of [more patients], just to make sure it is done. But then I get frustrated about the shift schedule | 15 |
| Organi-sational | Job demands | High workload associated with lack of time for participation in health promotion | […] that I am basically just rotating, jumping [from patient to patient]. And when the shift is done […] I am coming home […] and realize how tired I am. You’re just done. | 7 |
|  | Occupational health promotion logistics | Inability to participate in occupational health promotion due to lack of time/lack of personnel/ill-scheduled time windows of courses | There are [occupational health promotion programs], but they are relatively uninteresting. The offers usually are not available continuously but only at a certain time and date, and when you work shifts irregularly, you can’t attend the courses. You can’t join every time but you need some consistency for these kind of things and this is just not given. | 22 |
|  | Occupational health promotion attractivity | Low personal relatedness toward occupational health promotion offers | I don’t use any of them […]. I don’t think these courses are any good to cope with work stress. | 9 |
|  | Work site-residence distance | High distance between working site and home residence as reason for inability to participate in occupational health promotion | As I work full-time, I usually don’t have the energy for exercising after my shifts, as my way home takes a long time | 5 |

| A.2. Example statements for each magnitude (1-5) per health promotion determinant (translated) | | | | | |
| --- | --- | --- | --- | --- | --- |
| Health promotion determinant (respective interview question) | Example statements per magnitude | | | | |
|  | very low/negative | low/rather negative | moderate/neutral | high/rather positive | very high/positive |
| **Self-efficacy** (How do you estimate your personal confidence to perform health behaviors in the future?) | I think I cannot change anymore, I won't be able to do it any better | Because of my full-time employment, I usually do not have the power for exercising after arriving at home, as I also have a long commute | Yeah, well, at some point maybe I should do something for myself. One gets older, but I have not really made up my mind about it. I don't know | I am pretty confident | [very good] because I am in a phase in my life where I personally think that my health has another valence than for a 30 year old |
| **Outcome expectancies** (What would change for you personally if you participated in health promotion programs?) | I think I would be more stressed | I believe I would, or I don't know, haven't tried it yet, but it wouldn't help me if my employer would offer me sport courses, relaxation techniques or the like | It probably would not be bad. Yeah, perhaps it helps with relaxing. I can't really tell. I never participated in those things | Perhaps I would be able to better cope with stress | […] important with compensational exercising in any way. For the mind and for the body. And since I continuously exercise, I definitely feel improvements in terms of general health |
| **Current health promotion activitiy** (Have you lately done something for your health? – If so: ‘What health behaviors have you engaged in? How often per week? How long per unit?) | Not really | I go for walks in nature [1-2 x / week, up to 180 minutes] | Yeah, I walk the dog [3-4 times / day, 15-60 minutes], I do not do sports […] But anyway I am a very mobile person, because I always have energy and power. And I am moving the whole day. Therefore, I think, I do not need any sports | I run about 3-4 times per week of which one is usually a longer run, about 11-12 kilometers. So, about one hour. The other times I usually run for about half an hour | I do sports regularly. I go to the fitness club [1-2 x / week for 90-120 minutes], I run [1-2 x / week], play basketball [up to 3 x / week]. I usually try to cook fresh and healthy [...] I am big fan of head hygiene, for example reading which I do a lot |

| Table B1. Spearman Correlation matrix | | | | | | | | | |
| --- | --- | --- | --- | --- | --- | --- | --- | --- | --- |
| Variables | 1 | 2 | 3 | 4 | 5 | 6 | 7 | 8 | 9 |
| 1. SSCS raw score | 1 |  |  |  |  |  |  |  |  |
| 2. G Pattern score | -.114 | 1 |  |  |  |  |  |  |  |
| 3. S Pattern score | -.469** | - | 1 |  |  |  |  |  |  |
| 4. A Pattern score | .312* | - | - | 1 |  |  |  |  |  |
| 5. Resource Frequency | -.13 | .243 | .21 | -.233 | 1 |  |  |  |  |
| 6. Barrier Frequency | .199 | -.332* | -.073 | .107 | -.337* | 1 |  |  |  |
| 7. Self-efficacy | -.187 | .057 | .142 | -.219 | .517** | -.115 | 1 |  |  |
| 8. Outcome expectancies | .182 | .218 | -.089 | .049 | .238 | .153 | .237 | 1 |  |
| 9. Health behavior | -.238 | .124 | .185 | -.315* | .452** | -.204 | .691** | .218 | 1 |
| *: p < .05; **: p < .01 | | | | | | | | | |
